# All-trans retinoic acid in solid-tumour immunity: context-dependent signalling, cellular states, and combination therapy

**DOI:** 10.3389/fimmu.2026.1953814

**Published:** 2026-10-20

**Authors:** Yikai Han, Feng Wang, Taiying Lu

**Affiliations:** Department of Oncology, The First Affiliated Hospital of Zhengzhou University, Zhengzhou, China

**Keywords:** all-trans retinoic acid, cancer immunotherapy, combination therapy, myeloid-derived suppressor cells, retinoic acid signalling, tumour microenvironment

## Abstract

All-trans retinoic acid (ATRA) influences several processes relevant to cancer immunotherapy, yet its effects in solid tumours range from relief of myeloid suppression to reinforcement of immune tolerance. This divergence reflects the source and duration of retinoid exposure, the molecular state of responding cells and the phase of treatment. We examine these determinants across myeloid, tumour-cell, lymphoid and stromal compartments, distinguishing administered ATRA and ATRA-containing formulations from locally produced retinoic acid (RA) and interventions targeting the RA pathway. Local synthesis and CYP26-mediated catabolism regulate availability, whereas intracellular binding proteins, RAR/RXR complexes, transcriptional coregulators and chromatin influence the response to that exposure. Interactions with interferon, TGF-β, IL-10 and PD-L1 further connect retinoid signalling to immune recognition, regulation and adaptive resistance. Experimental studies support distinct combination strategies based on myeloid differentiation, tumour-cell recognition, preservation of effector function and stromal reprogramming. Human immunotherapy studies show pharmacodynamic activity and combination feasibility, with mixed clinical results across uncontrolled and comparative cohorts. We propose that translation should prioritise a defined, reversible barrier to immunity, pair blood measurements with functional tumour sampling, and compare ATRA-containing treatment with the same partner regimen alone. This approach gives context dependence a testable basis and directs ATRA development towards biologically selected combinations.

## 1 Introduction

All-trans retinoic acid (ATRA) established differentiation therapy as a successful treatment strategy in acute promyelocytic leukaemia. Its application to solid tumours raises a different question: can a retinoid change the malignant and non-malignant cells that determine whether an antitumour immune response succeeds? (1, 2) Early experiments showed that ATRA reduced suppressive immature myeloid populations and improved vaccination in tumour-bearing mice; subsequent work linked differentiation to glutathione accumulation and reduced reactive oxygen species (3, 4). Patient studies then connected ATRA exposure with circulating myeloid differentiation and antigen-specific immune responses (5, 6). These findings established an immunological rationale beyond direct inhibition of tumour growth.

The immunological rationale for ATRA now encompasses responses across tumour and microenvironmental compartments (7, 8), including tumour-cell interferon (IFN) responses and antigen presentation (9), recognition ligands (10) and stromal organisation (11). At the same time, tumour-derived retinoic acid (RA) can promote suppressive monocyte differentiation, and autocrine RA synthesis can restrain dendritic-cell vaccine activity (12, 13). The coexistence of these effects challenges a general immune-stimulatory model of ATRA. It instead places the source of the signal and the state of the recipient cell at the centre of therapeutic interpretation. **Figure 1** summarises this evolution in the field.

**Figure 1:**
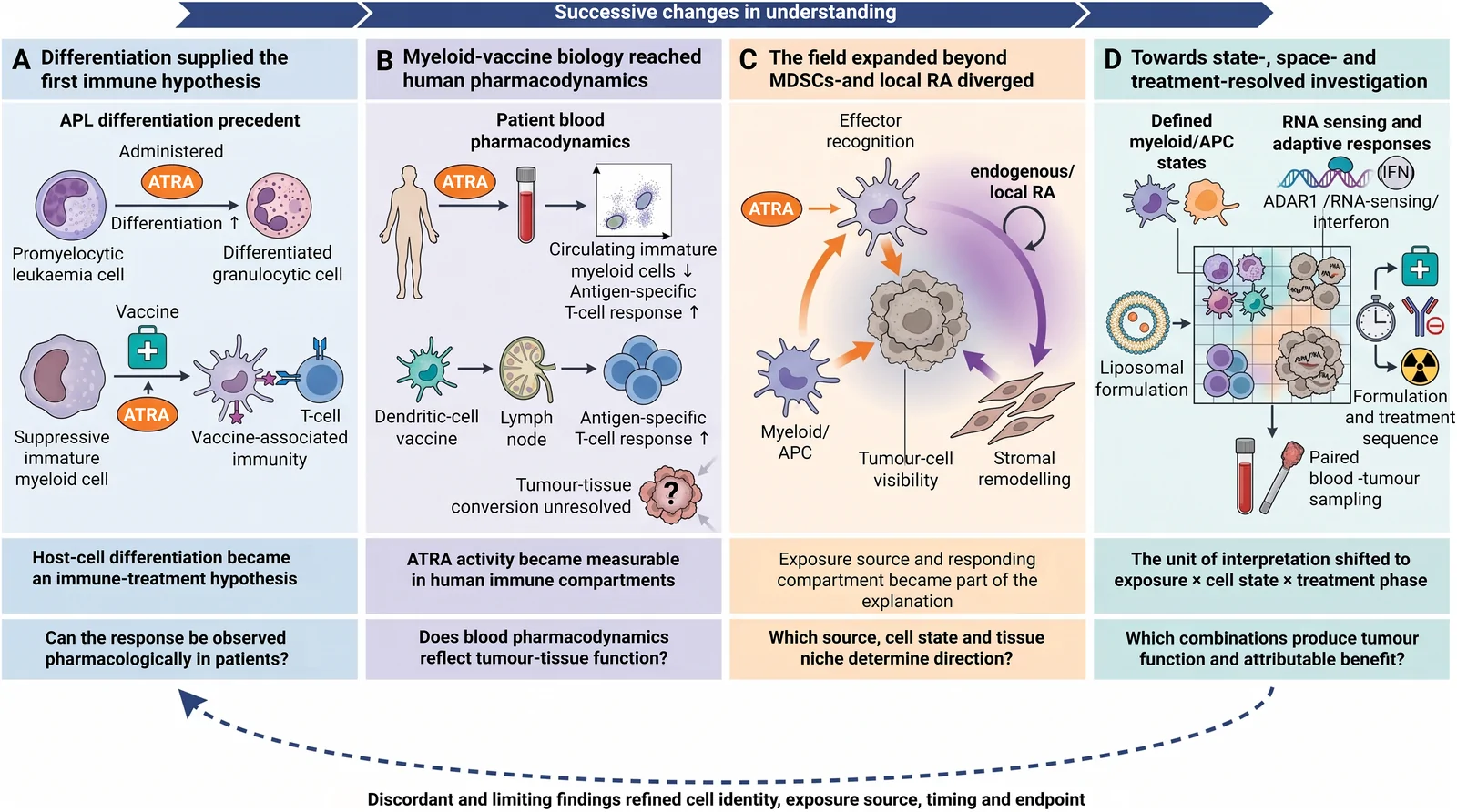
Evolution of the conceptual framework for ATRA in solid-tumour immunity. **(A)** The acute promyelocytic leukaemia differentiation precedent generated the hypothesis that administered ATRA could promote differentiation of suppressive immature myeloid cells and thereby enable vaccine-associated immunity. **(B)** The myeloid–vaccine programme extended this concept into patients through circulating myeloid pharmacodynamics and antigen-specific immune responses. **(C)** Expansion to effector recognition, tumour-cell visibility and stromal remodelling, together with studies of endogenous or local retinoic acid, showed that exposure source and responding compartment influence biological direction. **(D)** Contemporary research increasingly resolves cell state, RNA sensing and adaptive responses, formulation and treatment sequence, and paired blood–tumour sampling. The upper arrow traces these shifts in understanding; the dashed return arrow shows how discordant findings prompted closer examination of cell identity, exposure source, timing and functional endpoints. Created in BioRender. Han, Y. (2026) https://BioRender.com/o2fh3y2.

Extending differentiation therapy to solid tumours requires identifying the malignant or host-cell population whose response contributes to treatment activity. A treatment can restrain malignant-cell proliferation while favouring regulatory immune cells, or improve antigen presentation without overcoming a separate defect in effector recognition. Conversely, a drug with modest direct cytotoxic activity may become useful when it removes a host-cell constraint on vaccination or checkpoint therapy. The early myeloid studies and subsequent tumour-derived RA studies make this distinction experimentally tangible: they manipulate different sources of retinoid exposure and measure different cellular transitions (3, 4, 12, 13). The responding cell and its treatment environment therefore provide the basis for selecting a retinoid intervention.

Three developments make a renewed synthesis useful. Molecular studies now connect retinoid availability to receptor regulation, intracellular ligand handling and post-translational effects (14, 15). Immune studies increasingly distinguish suppression, recruitment, antigen recognition and persistence of effector function (16). Human programmes have generated both pharmacodynamic signals and longer clinical follow-up (17, 18), alongside less favourable results in other treatment settings (19). These developments allow the field to move beyond cataloguing positive combinations towards explaining which component of immunity changes, why that change occurs and whether a partner treatment can use it.

This narrative Review examines the mechanisms underlying divergent retinoid responses and their implications for combination immunotherapy. We distinguish four interventions: conventional administered ATRA; formulation-defined ATRA, whose carrier or co-delivered agent contributes to activity; endogenous or locally produced RA; and perturbation of RA synthesis, catabolism or receptors. Experimental studies and clinical reports provide the principal evidence, with published conference abstracts and trial registrations identified separately. Non-tumour immune studies are included where they explain molecular mechanisms relevant to the responding cell.

The exposure–cell-state–treatment-phase framework can be expressed as six questions. Which cells are exposed, and which show target engagement? What concentration and duration of exposure are achieved? Which receptors, binding proteins and transcriptional states are present in those cells? Does exposure occur during antigen priming, effector expansion, established suppression or treatment-induced tissue injury? Where are the responsive cells relative to tumour cells and lymphocytes? Finally, does the measured endpoint capture a molecular change, an immune function, immune-dependent tumour control or a clinical outcome? These questions connect the molecular mechanisms discussed below with the design and interpretation of treatment studies.

## 2 Molecular determinants of retinoid responses

### 2.1 Local synthesis, catabolism and achieved exposure

Retinoid availability is regulated before receptor engagement. Retinol is converted through retinaldehyde to RA, with ALDH1A enzymes catalysing the final oxidation. ALDH1A2-dependent synthesis can generate an autocrine signal within an antigen-presenting cell or a paracrine signal between neighbouring cells. CYP26 enzymes metabolise RA and constrain its concentration and duration (14, 20, 21). Local synthesis therefore creates exposure patterns that systemic dosing may not reproduce. Identifying RA-producing cells and identifying the cells that respond to RA are complementary requirements for understanding a tissue response.

Tumour studies illustrate the functional importance of this spatial distinction. Devalaraja and colleagues showed that tumour-derived RA directed intratumoural monocytes towards an immunosuppressive state (12). Fang and colleagues identified an autocrine ALDH1A2–RA circuit that limited dendritic-cell vaccine efficacy; inhibiting ALDH1A2 improved vaccine activity in the tested models (13). Conversely, restoration of deficient intestinal RA supported protective CD8-positive T-cell immunity and limited inflammation-associated tumour development in colorectal models (22, 23). Together, these findings indicate that the consequence of changing RA availability depends on the circuit being altered: restoring a deficient mucosal signal and interrupting a tolerogenic antigen-presenting-cell circuit have different biological objectives.

Isoform-selective inhibition now gives this distinction a pharmacological basis. RALDH1 inhibitors reduced RA production in human hepatocellular-carcinoma cells and restrained tumour growth in mouse models, accompanied by fewer tumour-supporting macrophages and increased intratumoural T-cell activity (24). An ALDH1A3 drug-development study identified retinoid-insensitive RA-producing cancer cells that continued to signal to neighbouring immune cells. ALDH1A3 inhibition interrupted this paracrine pathway, and orally administered MBE1.5 produced antitumour activity in mice (25). The therapeutic target is therefore the source–recipient relationship: an ALDH-high tumour can supply an immunoregulatory signal even when its malignant cells respond poorly to additional ligand.

Administered ATRA adds systemic pharmacokinetics to this local regulation. Oral exposure is variable, and induction of retinoid metabolism can reduce exposure during treatment. Breast-cancer experiments demonstrated autoinduction of metabolism in RA-sensitive cells, while pharmacokinetic modelling links systemic exposure to metabolic clearance and drug interactions (26, 27). CYP26 expression can thus represent both a determinant of baseline exposure and a response to receptor activation. Serial measurements during a defined dosing interval would distinguish pre-existing clearance capacity from treatment-induced feedback.

These pharmacological processes complicate the interpretation of dose–response relationships. A dose reported in an animal experiment, a concentration maintained in cell culture and an oral clinical dose describe different exposures. Persistent culture exposure may favour transcriptional differentiation, whereas intermittent dosing may principally affect a restricted activation or treatment window. The renal-cell carcinoma pharmacodynamic study is instructive because it related achieved ATRA exposure to immune measurements, while historical solid-tumour studies explored conventional and liposomal schedules with differing pharmacokinetic behaviour (5, 28–30). Relating dose to target-cell exposure and receptor engagement would connect these pharmacokinetic differences to the resulting immune response.

Formulations can alter solubility, distribution, release and the cells reached by ATRA. Nanoparticles that co-deliver immune agonists or suppress CYP26A1 address different pharmacological constraints from conventional oral treatment (31–34). Comparisons with free ATRA, empty carrier and partner cargo resolve their respective contributions. For immunological applications, the useful delivery endpoint is sustained activity in the intended cell population, which may differ from either plasma exposure or total tumour accumulation.

### 2.2 RAR/RXR transcription, coregulators and chromatin

The canonical transcriptional response involves retinoic acid receptors (RARα, RARβ and RARγ), which form heterodimers with retinoid X receptors (RXRs) and regulate retinoic acid response elements. ATRA binds primarily to the RAR partner. In the classical model, unliganded receptors recruit corepressors such as NCOR1 or SMRT/NCOR2 and histone deacetylase-containing complexes. Ligand binding favours recruitment of coactivators, including histone acetyltransferase-associated machinery, permitting transcription at responsive loci (14, 20, 35). Receptor isoforms, cooperating transcription factors and chromatin accessibility determine which genes can respond within a particular cell.

Transcriptional repression of RARβ in breast-cancer cells provides a mechanistic example. Disrupting the interaction between SIN3A and the MAD1/MXD1 repression domain increased RAR expression and reporter activity, with additional activation by an RARα-selective agonist. Promoter and chromatin-immunoprecipitation experiments implicated E-box-associated SIN3A repression alongside classical receptor-associated regulation (35). These experiments identify transcriptional repression as a determinant of retinoid responsiveness and support combining ligand exposure with restoration of receptor expression.

Chromatin also links receptor activity to immune-cell identity. Combined RXRα/β deletion in mouse serous-cavity macrophages altered chromatin accessibility and macrophage identity and reduced ovarian-tumour progression (36). RXRs partner with several nuclear receptors, placing this macrophage phenotype within a broader network of nuclear-receptor regulation. Retinoid–epigenetic interactions in breast cancer likewise support cell-specific regulation (37). Receptor activity, accessible chromatin and target-gene induction together describe how ligand exposure is translated into a cell-specific transcriptional response.

Direct tumour studies connect this chromatin regulation to immune function. In mouse skin, LSD1 inhibition increased H3K4 methylation at AP-1-enriched retinoid and immune regulatory regions, enhanced dendritic-cell recruitment and restricted squamous-cell carcinoma growth. RAR blockade partly reversed tumour restriction, whereas CD4-positive T-cell depletion abolished it, placing retinoid signalling within a broader epithelial immune response (38). In colorectal-tumour macrophages, tumour-derived lactate increased H3K18 lactylation and repressed RARγ. Loss of RARγ-mediated restraint on TRAF6–NF-κB signalling increased macrophage IL-6 production, which promoted STAT3 signalling in colorectal-cancer cells (39). These studies link environmental metabolites and chromatin regulation to receptor competence and to communication between malignant and immune cells.

Differentiation competence and current activation state jointly determine the response to retinoids. A cell may express an RAR yet retain a repressed differentiation programme; another may possess accessible response elements but encounter cytokines that redirect the downstream response. The SIN3A experiments illustrate the former problem, and the TGF-β-dependent lymphocyte experiments discussed below illustrate the latter (35, 40). Receptor abundance, chromatin accessibility and inflammatory signalling therefore answer complementary questions. For biomarker development, baseline transcriptional profiles describe lineage and prior signalling, whereas changes after retinoid exposure reveal the inducibility of the intended immune function.

The macrophage RXR-deletion findings also raise a practical question about receptor-directed treatment. Removing a receptor partner changes the transcriptional system more broadly than adding an RAR ligand, and receptor-selective agonism introduces another pharmacological comparison (36, 41). Comparing these interventions in the same cellular state can identify the receptor activities responsible for maintaining it. Chromatin accessibility and target-gene induction provide a mechanistic basis for selecting a compound capable of redirecting that state.

### 2.3 Intracellular retinoid routing and non-canonical mechanisms

Intracellular binding proteins influence the destination of RA. CRABP2 can deliver RA to nuclear RARs, whereas CRABP1 contributes to intracellular handling and non-canonical signalling (14, 20, 21). Schug and colleagues demonstrated that CRABP2 favoured RAR-dependent responses, while FABP5 directed RA towards PPARβ/δ-associated survival programmes in the experimental systems studied (42). The relative availability of these proteins therefore offers a mechanism for different outputs from the same ligand. This routing mechanism provides a candidate basis for selecting retinoid-responsive cellular states in immunotherapy.

Pancreatic-cancer experiments provide a direct test of the functional importance of intracellular routing. Gupta and colleagues examined patient-derived, low-passage pancreatic ductal adenocarcinoma lines and found contrasting responses associated with CRABP2 and FABP5 expression. FABP5-high, CRABP2-deficient cells resisted ATRA-mediated growth inhibition and showed increased migration or invasion, whereas FABP5-negative, CRABP2-expressing cells were more sensitive. Ectopic CRABP2 expression altered the resistant phenotype, and chromatin-modifying treatment restored endogenous CRABP2 expression in selected lines (43). Restoration of CRABP2 thus linked intracellular ligand routing to malignant-cell behaviour and xenograft growth. In pancreatic therapy, this epithelial response complements the stromal response to ATRA: intracellular routing in the cancer cell and quiescence in the stellate cell may jointly influence the outcome of systemic treatment.

Retinoid activity also extends beyond conventional nuclear transcription. Nuclear and extranuclear RARα participate in a regulatory circuit controlling T-cell activation and function, linking receptor signalling to the activation state of the lymphocyte (44). In pancreatic-cancer models, ATRA destabilised ADAR1 through retinoylation-associated dissociation of the deubiquitinase USP7 and enhanced immunotherapy (15). This post-translational route connects ATRA to RNA sensing and broadens the mechanisms that may underlie a treatment response. Receptor perturbation and protein-level rescue experiments can discriminate transcriptional and post-translational contributions within the same cellular system.

### 2.4 Interaction with IFN, TGF-β, IL-10 and PD-L1

Inflammatory signals help determine whether retinoid responses favour immune recognition or regulation. ATRA stimulated viral-mimicry-associated transcription, IFN responses and antigen-presentation programmes in breast-cancer cells (9). In IFN-γ-resistant colorectal-cancer cells, ATRA restored MGAT3 expression, increased bisecting N-glycosylation and stabilised IFN-γ receptor α, recovering receptor signalling (45). Other tumour models linked inflammatory states to checkpoint sensitivity, while the pancreatic ADAR1 mechanism connects retinoid exposure to RNA sensing (15, 46). These routes converge on immune recognition through different molecular processes. Their therapeutic consequence depends on subsequent antigen presentation, chemokine secretion and susceptibility to immune killing.

A complementary colorectal study connected endogenous retinoid metabolism with cytokine responsiveness. Integrated molecular profiling identified a lncRNA 60967.1–PLCD4-associated programme. In colorectal-cancer cells, lncRNA overexpression increased PLCD4, ATRA and IFNGR1 and enhanced IFN-γ-induced apoptosis. Separate experiments showed that exogenous ATRA increased IFNGR1 expression; the study also reported improved anti-PD-1 activity after lncRNA overexpression in a CT26 tumour model (47). These findings associate genetically altered retinoid metabolism with cytokine responsiveness, whereas the MGAT3 study directly links receptor glycosylation to IFN-γ signalling (45). Together, they identify retinoid availability and receptor processing as distinct points at which a cancer cell’s response to an effector-derived cytokine can change.

PD-L1 induction illustrates why an inflammatory response can have competing consequences. In gastric-cancer experiments, ATRA increased PD-L1, reduced killing by activated T cells and attenuated anti-PD-L1 treatment efficacy *in vivo*; ruxolitinib counteracted these effects (48). By contrast, the pancreatic ADAR1 study reported improved checkpoint treatment despite concurrent PD-L1 induction (15). PD-L1 induction therefore accompanies divergent checkpoint responses, with the therapeutic outcome shaped by concurrent changes in tumour recognition and effector activity.

TGF-β redirects RA responses during CD4-positive T-cell differentiation. RA increased Smad3 expression and phosphorylation, enhanced TGF-β-dependent Foxp3 induction and restrained Th17 differentiation, including through reduced IL-6 and IL-23 receptor expression. However, the increase in regulatory conversion observed *in vitro* was not reproduced as the same change in regulatory T-cell frequency during ongoing experimental autoimmune inflammation (40). The contrast between differentiating cells and ongoing inflammation implicates the surrounding cytokine environment in the persistence of the regulatory phenotype.

IL-10 regulation further separates distinct tolerogenic programmes. ATRA enhanced TGF-β-dependent Foxp3 induction but inhibited Il10 induction in developing mouse CD4-positive T cells (49). By contrast, RA-conditioned human dendritic cells induced suppressive, gut-homing IL-10-producing T cells; added TGF-β favoured Foxp3-positive cells over IL-10-producing cells (50). The contrasting regulatory outputs arise in different species, antigen-presenting-cell contexts and stimulation conditions, consistent with recipient-cell and cytokine-dependent regulation. IL-10 production, Foxp3 expression and suppressive function consequently represent distinct endpoints. **Table 1** compares representative molecular determinants and their experimental settings; **Figure 2** integrates them into the exposure–response framework.

**Table 1:** Molecular determinants of retinoid responses: experimental findings and interpretation.

Molecular determinant	Experimental model	Intervention and observed effect	Interpretation	Refs.
Local RA synthesis	Tumour-associated monocytes; dendritic-cell vaccines	Tumour-derived RA promoted suppressive monocyte differentiation; ALDH1A2 inhibition improved dendritic-cell vaccine activity.	Local synthesis may sustain a suppressive myeloid or dendritic-cell state.	(12, 13)
Catabolism and exposure	RA-sensitive breast-cancer cells; pharmacokinetic models	Retinoid exposure induced metabolism; clearance and drug interactions altered systemic exposure.	Achieved exposure reflects metabolic capacity and treatment-induced clearance.	(26, 27)
Transcriptional coregulators	Breast-cancer cells	Disruption of SIN3A–MAD1/MXD1 repression increased RAR expression and reporter activity; an RARα agonist further enhanced activity.	Corepressor activity can restrict retinoid-responsive transcription.	(35)
Receptors and chromatin	Mouse serous-cavity macrophages; ovarian-tumour model	Combined RXRα/β deletion altered accessibility and macrophage identity and reduced tumour progression.	RXR-dependent macrophage identity supported ovarian-tumour progression.	(36)
Chromatin–immune coupling	Mouse epidermis and squamous-cell carcinoma	LSD1 inhibition activated retinoid/AP-1 loci and restricted tumours; RAR blockade partly reversed the effect and CD4 depletion abolished it.	Retinoid-responsive chromatin contributes to epithelial immune competence.	(38)
Ligand routing	HaCaT keratinocytes; MCF-7 and NaF mammary tumour cells	CRABP2 favoured RAR responses; FABP5 directed RA towards PPARβ/δ-associated survival programmes.	Intracellular ligand routing can favour growth inhibition or survival.	(42)
Post-translational regulation	Pancreatic-cancer models	ATRA-induced ADAR1 retinoylation disrupted USP7 association, destabilised ADAR1 and enhanced immunotherapy.	ADAR1 destabilisation links ATRA to RNA sensing beyond canonical RAR transcription.	(15)
Cytokine context	Mouse CD4 T cells; RA-conditioned human dendritic cells	RA enhanced TGF-β-dependent Foxp3 induction. IL-10 responses differed between developing T cells and dendritic-cell-mediated induction.	Foxp3 induction and IL-10 production are distinct, context-dependent regulatory responses.	(40, 49, 50)
Checkpoint feedback	Gastric- and pancreatic-cancer models	ATRA reduced activated T-cell killing and anti-PD-L1 efficacy in gastric models; checkpoint therapy improved in pancreatic models despite PD-L1 induction.	Checkpoint outcome depends on the accompanying tumour-cell and effector response.	(15, 48)

Representative mechanisms are shown with the model, intervention and measured response. Receptor deletion and synthesis inhibition alter endogenous signalling, whereas administered ATRA changes ligand exposure. RA, retinoic acid; ATRA, all-trans retinoic acid; RAR, retinoic acid receptor; RXR, retinoid X receptor; CRABP2, cellular retinoic acid-binding protein 2; FABP5, fatty acid-binding protein 5; PPARβ/δ, peroxisome proliferator-activated receptor β/δ; ADAR1, adenosine deaminase acting on RNA 1; USP7, ubiquitin-specific peptidase 7; TGF-β, transforming growth factor β; IL-10, interleukin-10; PD-L1, programmed death-ligand 1.

**Figure 2:**
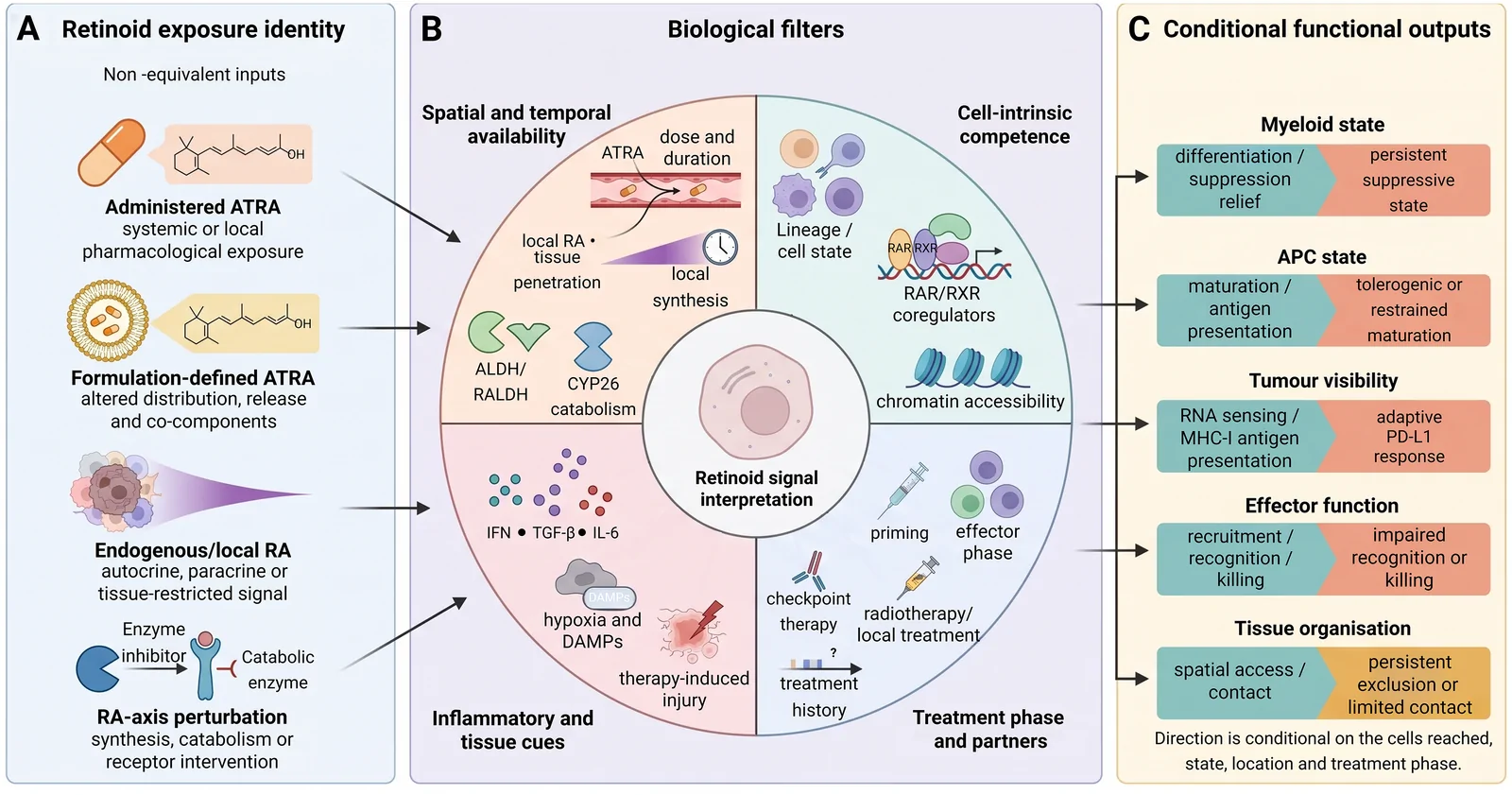
Retinoid exposure, biological context and conditional immune outcomes. **(A)** Administered ATRA, formulation-defined ATRA, endogenous/local RA and RA-axis perturbation are non-equivalent inputs. **(B)** Spatial and temporal availability, cell-intrinsic competence, inflammatory and tissue cues, and treatment phase and partners influence retinoid signal interpretation. **(C)** Conditional outcomes are illustrated for myeloid and antigen-presenting cells, tumour visibility, effector function and tissue organisation. Arrows connect retinoid exposures to the biological factors that shape their effects and the resulting functional outcomes. The question mark beside treatment history denotes the uncertain influence of prior clinical treatment on subsequent retinoid responsiveness. ATRA, all-trans retinoic acid; RA, retinoic acid; ALDH, aldehyde dehydrogenase; RALDH, retinaldehyde dehydrogenase; CYP26, cytochrome P450 family 26; RAR, retinoic acid receptor; RXR, retinoid X receptor; IFN, interferon; TGF-β, transforming growth factor β; IL-6, interleukin-6; DAMPs, damage-associated molecular patterns; APC, antigen-presenting cell; MHC-I, major histocompatibility complex class I; PD-L1, programmed death-ligand 1 (14, 20, 21, 35, 36, 40, 42, 49, 50). Created in BioRender. Han, Y. (2026) https://BioRender.com/rcgeqp7.

The interaction between retinoid handling and cytokine signalling offers a molecular explanation for state-dependent responses. CRABP2 restoration changes the intracellular destination of the ligand, whereas MGAT3 restoration changes the stability of the receptor through which a cancer cell receives IFN-γ (43, 45). These interventions act at different points in the response: one alters retinoid signalling itself, and the other alters sensitivity to an immune effector signal. The TGF-β and IL-10 experiments add a third determinant, in which the surrounding cytokines shape the differentiation programme induced by RA (40, 49, 50). Taken together, these findings suggest that a useful molecular profile would combine retinoid availability, receptor competence and the dominant cytokine response. This integrated view also identifies distinct opportunities for intervention: improve ligand delivery where exposure is limiting, restore transcriptional competence where the response programme is repressed, or choose a partner therapy that uses the immune function induced by ATRA.

## 3 Cellular mechanisms of ATRA-mediated tumour immunity

### 3.1 Myeloid differentiation and antigen-presenting cells

Suppressive immature myeloid cells were the first non-malignant target linked to ATRA-enhanced tumour immunity. In tumour-bearing mice, ATRA reduced these populations and improved vaccination. Subsequent work connected differentiation to glutathione accumulation and reduced reactive oxygen species, restored antigen-presenting capacity in defined settings and limited myeloid expansion induced by antiangiogenic treatment (3, 4, 51, 52). Myeloid differentiation in these models was coupled to recovery of antigen presentation and vaccine responsiveness.

Myeloid heterogeneity qualifies that differentiation model. Myeloid-derived suppressor cells (MDSCs) comprise suppressive cells of neutrophil and monocyte lineages; their identity, developmental origin and functional diversity remain active areas of investigation (53). In a mammary-tumour model, macrophages were more suppressive ex vivo than the phenotypically immature populations; cyclophosphamide-induced suppressive cells also differed from cells generated by tumour growth (54, 55). A decline in a broad myeloid population can therefore reflect differentiation, redistribution or selective loss, each with different functional consequences. Suppression assays provide the necessary link between phenotype and biological activity.

Metabolic reprogramming provides an additional myeloid mechanism. Sun and colleagues found reduced retinol-metabolism machinery and RA synthesis in human colorectal tumours. In a CD34-positive precursor model, exogenous RA reduced the generation of suppressive polymorphonuclear MDSCs with little effect on overall myeloid differentiation. The reported mechanism involved restrained glycolysis and AMPK activation; RA supplementation also reduced tumour growth and arginase-1-positive myeloid cells in colorectal mouse models (56). This study broadens the differentiation hypothesis by showing that suppressive function can change through metabolism without an equivalent shift in lineage differentiation. Together with the glutathione/ROS studies, it argues for analysing both the metabolic process sustaining suppression and the maturation state of the responding population (4).

Checkpoint-combination experiments support targeting particular suppressive states. ATRA-responsive granulocytic populations contributed to checkpoint resistance in LKB1-deficient non-small-cell lung cancer models (57). ATRA also reduced myeloid suppression and enhanced anti-PD-L1 treatment in cervical-cancer models, while studies involving Pin1 in colorectal cancer and MDSCs in hepatocellular carcinoma extended the range of candidate mechanisms (58–60). Recent conference reports describe treatment-associated neutrophil ageing, suggesting further heterogeneity within the responsive granulocytic population (61, 62). Retinoid sensitivity within these heterogeneous populations offers a functional basis for selecting myeloid-targeted combinations.

Macrophage and dendritic-cell studies reveal contrasting responses associated with activation state and the source of RA. Administered ATRA reduced tumour-promoting macrophage properties in prostate-cancer systems and promoted inflammatory macrophages in irradiated tumours (63, 64). Other studies documented dendritic-like differentiation, altered antigen presentation or migration to draining lymph nodes (65–67). In contrast, tumour-derived RA promoted suppressive monocyte differentiation, autocrine ALDH1A2 signalling limited dendritic-cell vaccine activity, and RARα blockade improved vaccination in a regulatory T-cell-associated model (12, 13, 68). Differentiating a suppressive host population and interrupting a tolerogenic circuit in a vaccine dendritic cell thus address different barriers to priming. Their apparently opposing interventions are consistent once the cellular target and source of RA are specified.

Local tumour-derived RA can also promote antigen presentation during treatment-induced cell death. Sun and colleagues found that ferroptotic tumour cells released ATRA, which induced macrophage CD38 through RARα and activated TFEB-dependent autophagy and MHC-II expression. CD38 silencing disrupted this response, and ferroptosis-directed treatment enhanced anti-PD-1 activity in mouse models (69). Alongside the suppressive monocyte programmes described above, this result identifies the condition of the producing tumour cell as a determinant of paracrine signalling. The relevant distinction is between a persistent suppressive niche and a cell-death-associated antigen-presenting response.

The contrast between RA deficiency in colorectal cancer and RA production in suppressive monocyte niches is especially informative (12, 56). Reduced synthesis in one compartment can coexist with a biologically active local source elsewhere, giving cellular localisation a central role in interpreting whole-tumour RA measurements. The same reasoning applies to a bulk ALDH1A2 or CYP26 signal, which combines expression from malignant and non-malignant cells. Combining spatial mapping with suppression assays would connect the RA-producing population to the functional response of neighbouring monocytes and identify suppressive states that are reversible by treatment.

The antigen-presenting response extends to other myeloid populations. A pan-cancer mast-cell study combined single-cell and spatial analyses with functional experiments showing that RA and IFN-γ increased CIITA-associated antigen-presentation machinery, CXCL16 secretion and T-cell activation through RARα (70). This cytokine-dependent response broadens the range of retinoid-responsive accessory cells and connects receptor activity with both recruitment and antigen presentation.

### 3.2 Tumour-cell recognition and adaptive immune resistance

Tumour cells provide a second route through which ATRA can alter immunity. In breast cancer, ATRA induced IFN-associated transcription and antigen presentation (9), and increased ICAM-1 expression (71). In cervical cancer, RA combined with IFNγ modulated major histocompatibility complex molecules and ICAM-1 (72). Sequential treatment of melanoma cells with ATRA and poly(I:C) altered chemokine and IFNβ secretion through TLR3- and MDA5-dependent processes (73). RA also induced IFN regulatory factor 1 through a STAT1-independent pathway in squamous carcinoma cells (74). These findings place retinoid-responsive tumour cells upstream of immune recruitment and recognition.

Recent time-series and chromatin analyses identified IRF1 as an early coordinator of ATRA-responsive transcription. IRF1 knockdown impaired differentiation and growth-control responses, and related signatures were enriched across ATRA-sensitive cancer contexts (75). The central perturbation experiments were performed in an APL model, with cross-cancer analyses extending the proposed response signature. These findings complement the earlier tumour-cell IFN studies by identifying an inducible transcriptional regulator for further evaluation in solid tumours.

These tumour-cell experiments address several consecutive requirements for immune attack. Antigen-presentation machinery determines whether a peptide can be displayed, adhesion molecules contribute to cell contact, and secreted chemokines influence recruitment. The breast-cancer viral-mimicry findings and the sequential ATRA–poly(I:C) experiments illuminate different parts of this sequence (9, 73, 74). The therapeutic consequence of an IFN response depends on how transcription is translated into surface antigen presentation and effector recognition. Comparing these steps in the same model would distinguish a presentation defect from checkpoint-mediated inhibition and guide the choice of partner therapy.

Specific molecular mechanisms strengthen this connection. ATRA-responsive OTUD6B programmes were linked to tumour suppression and immunotherapy in oesophageal squamous-cell carcinoma, and to CD8-positive T-cell recruitment through a DDX5/STAT3/CXCL11-associated mechanism in colorectal liver metastasis (76, 77). In pancreatic cancer, ADAR1 retinoylation and destabilisation connected ATRA to RNA sensing and improved checkpoint treatment (15). These mechanisms identify different points at which a tumour cell can become more accessible to immune attack, through cancer-specific molecular pathways.

Improved recognition can nevertheless be opposed by adaptive resistance. The divergent consequences of PD-L1 induction discussed in Section 2.4 exemplify this competition, as do changes in ligands recognised by natural killer (NK) cells (10, 15, 48). Differentiation may alter adhesion, antigen presentation, stress ligands and cell survival simultaneously. The therapeutic effect will depend on which of these changes limits killing by the relevant effector population. Functional killing assays and receptor blockade therefore help identify the appropriate partner therapy.

Repeated exposure can also alter subsequent immune recognition through transcriptional memory. In breast-cancer-derived metastatic tumoroids, oncogenic enhancer networks amplified the response to renewed RA exposure, sustained SOX9-associated quiescence and promoted escape from NK-cell surveillance (78). This finding gives exposure history a molecular meaning: an earlier signal changes how the same cell responds later. It also separates growth arrest from immune clearance, because a quiescent cancer cell can persist when its recognition by an effector cell falls. Duration and repetition of treatment therefore deserve analysis alongside the immediate differentiation response.

### 3.3 Lymphocyte activation, exhaustion and recognition

ATRA can influence lymphocytes through antigen-presenting cells and tumour-derived chemokines or through direct signalling within the lymphocyte. Topical ATRA inhibited melanoma partly through CD8-positive T-cell immunity, and ATRA–radiotherapy tumour control depended on adaptive immunity in the models studied (64, 79). RARα-dependent activation and cytokine regulation in human T cells, together with distinct nuclear and extranuclear receptor activities, provide a molecular basis for direct effects (44, 80). The relevant question is when retinoid signalling supports activation, expansion or maintenance of effector function.

Receptor abundance and ligand exposure can have distinct effects on effector differentiation. Niekamp and colleagues found that RARα overexpression restrained early cytotoxic T-cell differentiation and tumour entry, whereas RARα deficiency increased effector activity. The mechanism involved nuclear histone acetyltransferase activity, a TCF1-to-BATF transition and changes in trafficking receptors; RARα-deficient CAR T cells also controlled antigen-engineered mouse tumours more effectively. RA exposure reduced RARα protein and promoted BATF-dependent effector-gene expression in activated cells, providing a possible connection between ligand exposure and release of receptor-mediated restraint (81).

Kang and colleagues conditioned activated CD8-positive T cells with ATRA during expansion, then assessed their function under hypoxic tumour-cell co-culture without additional ATRA. Conditioning preserved effector function through a response involving canonical WNT/β-catenin signalling and the long, β-catenin-binding TCF-1 isoform. β-catenin silencing abrogated the preservation of cytokine production and cytotoxic function. ATRA-conditioned adoptive cells and oral ATRA improved antitumour responses in mouse glioma models, including anti-PD-1 combinations and a postoperative model (16). Complementary single-cell analyses associated RA-responsive and WNT-related programmes with response in anti-PD-1-treated patients. The treatment experiments thus identify a functional response to conditioning, with patient transcriptomic associations supporting its clinical investigation.

The comparison turns on both the intervention and the cellular state being measured. RARα deletion changes receptor abundance throughout differentiation, whereas activation-phase ATRA conditioning supplies ligand before subsequent exhaustion-promoting conditions. Niekamp and colleagues linked effector differentiation to reduced Tcf7 expression and increased BATF activity (81). In the conditioning study, total TCF-1 and its long isoform increased before exhaustion induction; after induction, the long isoform remained enriched while total TCF-1 was similar to the control (16). These findings identify potentially complementary therapeutic objectives: generating cytotoxic effectors and maintaining their function during sustained antigen exposure. A direct timing comparison could follow the differentiation and persistence of the same cells, relating receptor abundance and TCF-1 isoform composition to serial measurements of killing.

Retinoid signalling also regulates the persistence of lymphocytes within tissues. In mouse infection and adoptive-transfer experiments, Obers and colleagues identified an RA–RARα-dependent pathway for CD8-positive tissue-resident memory T-cell differentiation. RA supported a TGF-β-independent residency programme in the liver and cooperated with TGF-β in the small intestine. Blocking RA signalling promoted intestinal resident-cell migration to draining lymph nodes, linking local signalling to tissue retention (82). These findings extend the role of RA beyond initial recruitment to the maintenance of a local lymphocyte population. For tumour-directed treatment, they motivate testing how retinoid exposure affects retention alongside the effector function of cells that enter the tissue.

Retinoids can also favour regulatory lymphocyte responses. TGF-β and dendritic-cell-derived RA support tolerogenic programmes under defined conditions (40, 49, 50), contributing to the broader role of RA in immune homeostasis (83). Vitamin A-treated NK cells have been reported to reduce IFNγ production and support regulatory T-cell differentiation (84). For systemic ATRA, the balance between effector preservation and regulatory induction is likely to depend on which cells receive the signal during treatment. This makes functional measurements of both populations more informative than lymphocyte counts alone.

Innate-like lymphocytes provide another direct target. In nasopharyngeal carcinoma, serum RA was inversely associated with TIM-3 expression on circulating Vδ2 T cells. Functional experiments showed that RA/RAR signalling reduced TIM-3, increased NF-κB activity and enhanced effector cytokines; RA also improved the activity of transferred human Vδ2 cells in nasopharyngeal-carcinoma xenografts (85). The patient observations and intervention experiments thus converge on a defined lymphocyte population, extending retinoid-responsive effector regulation beyond conventional CD8-positive cells.

NK-cell studies distinguish recruitment from target recognition. ATRA-induced chemerin partly mediated CMKLR1-dependent NK-cell recruitment, while ATRA enhanced cytokine-induced killer-cell cytotoxicity against lung adenocarcinoma in association with MICA and IL-2 (86, 87). Conversely, ATRA reduced the susceptibility of a gastric-cancer line to lymphokine-activated killer cells (88). In oesophageal cancer stem-like cells, differentiation restored ULBP-1 expression and NK-cell killing (10). The target-ligand state can therefore determine whether an increase in effector recruitment produces effective cytotoxicity, a consideration that also applies to ATRA-containing NKG2D-CAR T-cell combinations (89).

Retinoid receptor agonism can additionally change the signals through which tumour cells engage NK cells. In prostate-cancer models, adapalene reprogrammed the senescence-associated secretory phenotype induced by docetaxel and enhanced NK-cell-mediated clearance, including activity of transferred human NK cells in xenografts (90). This distinct receptor-agonist strategy acts through the treated tumour-cell secretome, complementing target-ligand restoration and direct effector conditioning.

### 3.4 Stromal mechanics and spatial access

Stromal organisation influences whether effector cells reach malignant cells. Activated pancreatic stellate cells can sequester CD8-positive T cells away from the juxtatumoral compartment. In KPC mice, ATRA increased juxtatumoral CD8-positive T-cell numbers; migration assays linked preferential T-cell attraction towards activated stellate cells to CXCL12, with reduced attraction after ATRA treatment or CXCL12 knockdown (91). ATRA mechanically reprogrammed pancreatic stellate cells, reduced matrix remodelling and inhibited cancer-cell invasion (11). Formulation-defined approaches extend this rationale to hepatic stellate cells and colorectal stroma (92, 93). In fibrotic colorectal-tumour models, FAP-directed RA nanoparticles reduced fibroblast activation and collagen deposition and increased intratumoural oxaliplatin exposure, linking stromal reprogramming to drug delivery (93). Together, these studies identify soluble chemotactic cues and matrix mechanics as complementary targets of stromal reprogramming.

Translation of stromal reprogramming depends on how stellate-cell state, chemotactic signals and matrix mechanics jointly regulate access to tumour nests. Measuring these processes alongside tumour–T-cell contact would test whether reduced invasion and improved immune access reinforce one another or favour different treatment schedules. STARPAC established the feasibility of combining ATRA with chemotherapy and incorporated exploratory imaging and soluble biomarkers of stromal activity (94–96). These clinical measurements provide a starting point for connecting stromal pharmacodynamics to spatial immune function, as discussed in Section 5 and **Table 2**.

**Table 2:** ATRA-containing clinical regimens: pharmacodynamic findings, clinical outcomes and registered studies.

Study or regimen	Population and design	Observed finding or study status	Interpretation	Refs.
A. Human pharmacodynamic evidence				
ATRA → IL-2	RCC; 18 patients; sequential treatment	Exposure-related changes in circulating myeloid cells and ex vivo immune responses	Blood activity; subsequent IL-2 affects interpretation	(5)
Dendritic-cell vaccine + ATRA	SCLC; randomised initial stage with subsequent combination-arm expansion	p53-specific responses in 10/23 immune-evaluable patients versus 3/15 with vaccine alone	Antigen-specific activity; one linked study programme	(6, 112)
Ipilimumab + ATRA	Melanoma; small randomised pilot	Circulating MDSC and effector-cell measurements	Comparative peripheral pharmacodynamics; limited clinical precision	(113)
Pembrolizumab + ATRA	Melanoma; 24 treated, anti-PD-1-naive patients; single arm	Changes in circulating MDSCs and effector measurements	Blood-based biological response to the combination	(17)
STARPAC	Pancreatic cancer; 27 treated; ATRA + gemcitabine/nab-paclitaxel	ATRA pharmacokinetics, diffusion-weighted MRI and stromal/soluble markers	Exploratory stromal pharmacodynamics	(94–96)
B. Clinical outcomes				
Pembrolizumab + ATRA	24 anti-PD-1-naive patients; no concurrent control	ORR 71%; CR 50%; updated median PFS 13.8 months; five-year OS 54.7%	Single-arm regimen activity and durability	(17, 18)
KEYMAKER-U02A	Melanoma; 20 anti-PD-(L)1-refractory patients; conference abstract	ORR 0/20; prespecified expansion criterion not met	No responses in the refractory cohort	(19)
KEYMAKER-U02B	First-line melanoma; fave/pembro ± ATRA; 49/48 patients; abstract	ORR 33% in both arms; median follow-up 6.6 months	Same reported ORR at the early analysis	(120)
SCLC vaccine–ATRA	55 in initial randomised stage; 14 added to combination arm	No demonstrated improvement in principal salvage-response endpoint	Antigen-specific and clinical endpoints diverged	(112)
STARPAC	19 treated at recommended dose; 15 efficacy-evaluable	Median OS 11.7 months in the evaluable recommended-dose subset	Single-arm efficacy-evaluable subset	(94)
FOLFOX4 ± ATRA	HCC with extrahepatic metastases; double-blind RCT; 53/55 patients	OS HR 0.56 (95% CI 0.33–0.93); PFS HR 0.62 (0.41–0.94)	Randomised chemotherapy comparison; exploratory molecular analyses	(118)
C. Registered or incompletely reported studies				
STARPAC2	Randomised chemotherapy ± ATRA	Active, not recruiting; no registry results posted	Randomised design addresses the contribution of ATRA	(116, 117)
Additional registered studies	Other solid tumours and formulation-defined regimens	Registry details in S1; CRC safety and HF1K16 pharmacodynamic abstracts in S2	Published abstracts complement registry information	(119, 121–131)

Panels separate biological activity, patient outcomes and study registration; a study may inform more than one panel. Clinical outcomes refer to the complete regimen. Registry records checked 21 September 2026; published reports are identified separately. The initial melanoma PFS estimate was 20.3 months; 13.8 months is the updated estimate from the same cohort. RCC, renal-cell carcinoma; SCLC, small-cell lung cancer; IL-2, interleukin-2; MDSC, myeloid-derived suppressor cell; PK, pharmacokinetics; PD, pharmacodynamics; MRI, magnetic resonance imaging; ORR, objective response rate; CR, complete response; PFS, progression-free survival; OS, overall survival. Fave/pembro, favezelimab/pembrolizumab; HCC, hepatocellular carcinoma; RCT, randomised controlled trial; HR, hazard ratio; CI, confidence interval; MSS CRC, microsatellite-stable colorectal cancer. Further clinical detail is given in **Supplementary Table S2**.

Stromal selectivity becomes particularly important when cancer cells themselves have divergent retinoid responses. The CRABP2/FABP5 experiments in pancreatic cancer demonstrate why epithelial sensitivity should be measured alongside stromal pharmacodynamics (43). Paired assessment could identify tumours in which epithelial susceptibility and stromal access improve together, as well as settings in which selective delivery would preserve a favourable response in one compartment. This gives compartment-directed treatment a biological rationale beyond improving solubility or total tumour uptake. **Figure 3** summarises representative responses across the four cellular compartments.

**Figure 3:**
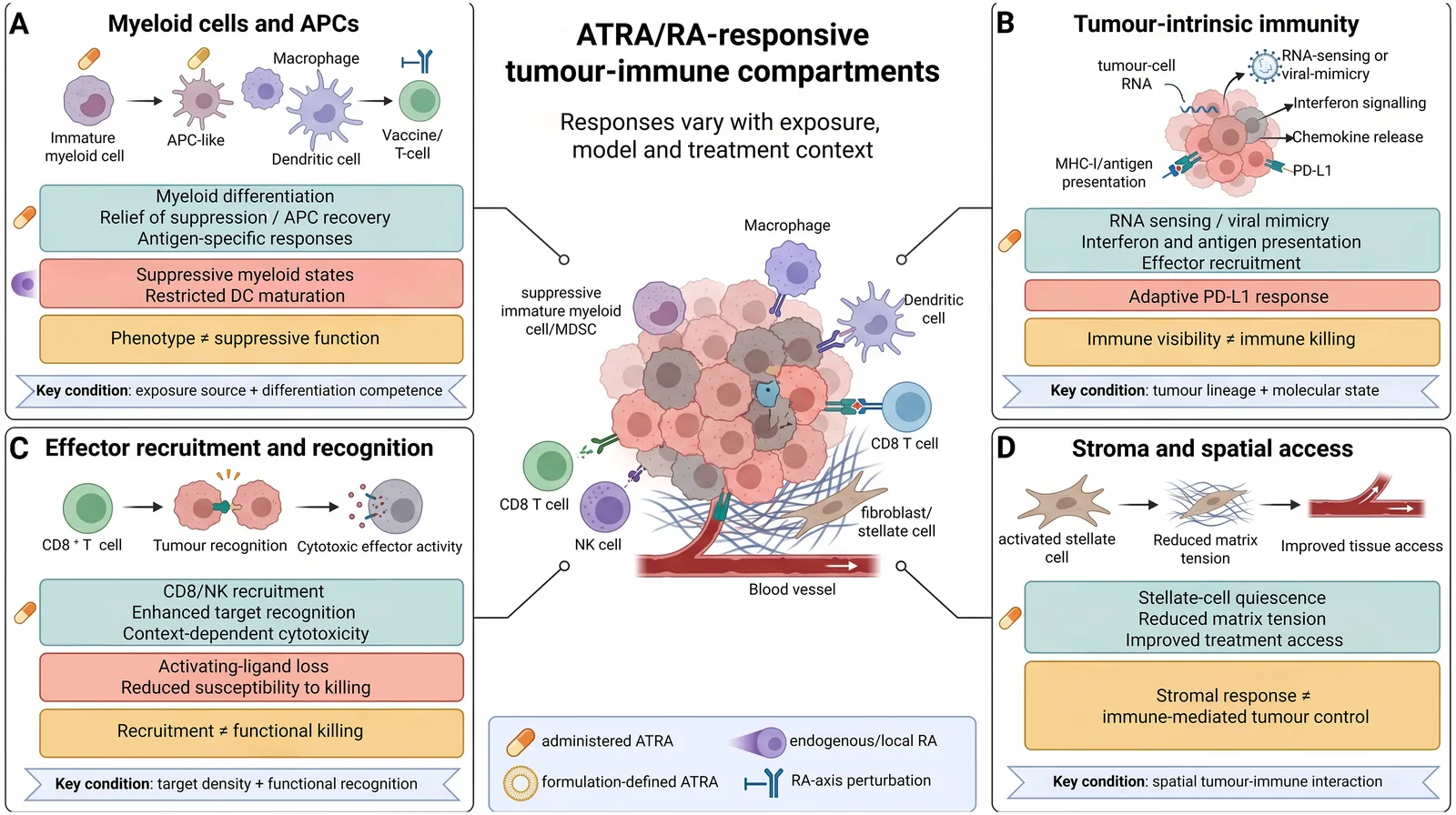
Context-dependent effects of ATRA/RA across tumour–immune compartments. **(A)** Myeloid differentiation, relief of suppression and antigen-presenting-cell recovery are contrasted with suppressive myeloid states and restricted dendritic-cell maturation (3, 4, 12, 13). Local RA can also promote macrophage antigen presentation following tumour-cell ferroptosis (69). **(B)** Tumour-intrinsic responses include RNA sensing, interferon and antigen-presentation programmes, effector recruitment and adaptive PD-L1 responses (9, 15, 48). **(C)** Recruitment, target recognition and cytotoxic function represent successive determinants of effector activity. Studies of NK, cytokine-induced killer and lymphokine-activated killer cells illustrate context-dependent changes in recruitment and susceptibility to killing (10, 86–88); ATRA conditioning can also preserve CD8-positive T-cell function under exhaustion-promoting conditions (16). **(D)** Stellate-cell quiescence and reduced matrix tension can improve treatment access (11). In KPC pancreatic-cancer mice, ATRA increased juxtatumoral CD8-positive T-cell numbers (91). Responses vary with retinoid exposure, model and treatment setting. Blue-green and coral boxes show potentially productive and counter-regulatory responses, respectively. Yellow boxes distinguish phenotypic or spatial changes from functional immune outcomes; ≠ marks the distinction between these levels of response. Exposure symbols identify the interventions or RA sources associated with selected findings. ATRA, all-trans retinoic acid; RA, retinoic acid; APC, antigen-presenting cell; DC, dendritic cell; MDSC, myeloid-derived suppressor cell; NK, natural killer; MHC-I, major histocompatibility complex class I; PD-L1, programmed death-ligand 1. Created in BioRender. Han, Y. (2026) https://BioRender.com/3ic5rxd.

## 4 Mechanism-guided combinations and treatment timing

### 4.1 Vaccination and checkpoint blockade

The rationale for a combination is strongest when the function modified by ATRA complements the function supplied by its partner. Vaccines supply antigen and priming; ATRA may relieve a suppressive host-myeloid state that constrains that response. Mouse vaccination studies support this complementarity (3, 97, 98). Scheduling experiments showed that the timing of ATRA relative to vaccination changes treatment activity, making the distinction between priming and effector expansion mechanistically relevant (99). Inhibiting an RA-dependent tolerogenic circuit within vaccine dendritic cells provides a separate way to improve priming (13, 68). The host population and vaccine cell are therefore distinct intervention targets. Combining host-myeloid differentiation with vaccine-cell-restricted RA-pathway inhibition is a testable strategy: its value would be established by improved antigen-specific responses accompanied by changes in both host suppression and vaccine-cell function.

Formulation-defined vaccines can coordinate retinoid exposure and immune stimulation anatomically. Co-encapsulated ATRA and CpG and ATRA-adjuvanted mRNA vaccination produced mucosal antitumour responses in colorectal models (32, 33). Carrier-dependent release coordinates the cells reached by ATRA with delivery of the immune stimulus, linking formulation design to antigen-specific responses.

Checkpoint blockade has at least three distinct ATRA rationales: relief of myeloid suppression (57, 58), improved tumour-cell recognition (15) and preservation of a responsive effector T-cell state (16). Each implies different pharmacodynamic measurements and sampling times. Suppression assays address the myeloid hypothesis; antigen presentation and killing address the tumour-cell hypothesis; sustained activation and function address the lymphocyte hypothesis. Receptor-selective compounds, including an RARγ agonist studied in lung cancer, probe related signalling with pharmacology distinct from ATRA (41). These mechanistic differences guide selection of both the combination and its biological endpoint.

Clinical selection follows the same logic. A tumour limited primarily by suppressive myeloid cells presents a different opportunity from one with insufficient antigen recognition or a depleted pool of functional effectors. These mechanisms can coexist, but their relative importance determines which measurement should guide a trial. Early enrichment studies could combine a baseline tissue phenotype with a short ex vivo ATRA-response assay, then test whether the anticipated cellular change occurs *in vivo*. This design first identifies pharmacodynamic responsiveness. Predictive value for the clinical benefit of adding ATRA would be assessed through a biomarker-by-treatment interaction in a controlled comparison.

Checkpoint combination selection should follow the functional consequence of tumour-cell priming for recognition and killing, as illustrated by the contrasting pancreatic and gastric responses (15, 48). Improved recognition accompanied by checkpoint-sensitive inhibition of killing would favour blockade. When recognition remains weak, a partner that restores antigen presentation, target-ligand density or effector availability may address the dominant constraint more directly. Tumour-cell priming and lymphocyte conditioning also imply different exposure windows: priming can be tested before effector engagement, whereas conditioning targets activation and expansion. Following target recognition and sustained killing under matched treatment conditions would identify which schedule converts the induced cellular response into greater antitumour activity.

### 4.2 Local treatment and cellular therapy

Radiotherapy changes the tissue context through injury signals, antigen release and myeloid recruitment. ATRA cooperated with irradiation to generate inflammatory macrophage states and improve immune-dependent local and distant tumour control in experimental models (64). Liposomal ATRA, cryo-thermal treatment, photodynamic vaccination and *in situ* vaccine systems extend this rationale (100–103). Their shared opportunity is to direct the post-injury response towards antigen presentation and effector entry. Comparing schedules at similar total exposure would identify when retinoid treatment best supports those processes.

Radiotherapy-associated host injury provides another context for retinoid intervention. Fu and colleagues studied radiation-induced astrocyte senescence and found that ATRA promoted elimination of senescent astrocytes, reduced their senescence-associated secretory phenotype and attenuated glioblastoma progression and radiation-related brain injury in experimental models (104). Conditioned-medium experiments connected the altered astrocyte secretome with glioma-cell behaviour. This is a host-tissue mechanism distinct from the inflammatory macrophage–T-cell interaction described above. After irradiation, retinoids may therefore act through inflammatory immune activation or modification of a tumour-supporting senescent-cell secretome, depending on the responding host population.

Cellular therapies offer a different degree of control over the responding population. ATRA-conditioned CD8-positive cells in glioma address effector state, whereas ULBP-1 restoration and NKG2D-CAR T-cell combinations address recognition in selected tumour systems (10, 16, 89). Long and colleagues found that ATRA reduced monocytic MDSCs and the suppressive activity of granulocytic MDSCs, improving GD2-directed CAR T-cell activity against sarcoma xenografts (105). This host-myeloid route complements direct effector conditioning and tumour-target modulation. Ex vivo conditioning permits exposure during a defined activation window followed by washout; systemic dosing also reaches antigen-presenting and stromal cells. A direct comparison could determine whether broader tissue effects reinforce or counteract the benefit of effector conditioning.

The anatomical site can determine which host mechanism limits cellular therapy. In pleural mesothelioma models, NF2 loss promoted accumulation of CRIg-positive suppressive macrophages through tumour-derived chemokines and RA, producing resistance to CAR T cells and anti-PD-1 therapy. The resistance phenotype differed from that in subcutaneous tumours, and interference with RA signalling restored CAR T-cell sensitivity in the tested setting (106). This site-dependent interaction connects tumour genotype, local retinoid signalling and macrophage state, supporting the use of anatomically relevant models when evaluating retinoid combinations.

These cellular-therapy approaches act at different points in the encounter between an effector and its target. Ex vivo conditioning changes the transferred cell; modulation of ULBP-1 or other recognition ligands changes the target; depletion or functional modification of suppressive myeloid cells changes the environment of that encounter (10, 16, 89, 105). Persistence of effector function, target-dependent killing and host suppression provide corresponding endpoints for comparing these approaches. The sarcoma xenograft work establishes a role for the host-myeloid route in that experimental system, while the glioma conditioning experiments support a direct lymphocyte route. Combining effector conditioning with myeloid modulation offers a testable strategy for sustaining transferred-cell activity within a suppressive tumour environment.

Local release kinetics can implement this sequence pharmacologically. A 2026 lipogel study co-delivered ATRA and doxorubicin with different sustained-release profiles to combine differentiation with immunogenic cell death in cancer stem-like populations. Local implantation enhanced checkpoint therapy, suppressed growth and metastasis, and reduced postoperative recurrence in mouse models (107). This formulation provides a direct test of coordinating the differentiation response with the subsequent immune stimulus within the same tissue.

Exposure duration is also a therapeutic choice. A transient conditioning pulse may be sufficient if it establishes a persistent functional state, whereas a continuously regenerated suppressive population may require repeated intervention. Conversely, prolonged systemic exposure could reach regulatory populations that were not the intended target. These alternatives favour experiments in which pulse, intermittent and sustained schedules are compared at defined exposure, with the relevant cellular response followed after drug withdrawal. Formulation-defined delivery is most persuasive when it demonstrably changes this duration or compartmental distribution and improves the corresponding immune function.

Treatment timing is therefore part of the mechanism of combination therapy. Its contribution is best tested within a fixed regimen, measuring the proposed cellular transition before the downstream immune outcome. Clinical differences between treatment-naive and refractory cohorts also reflect prior therapies, disease burden and partner agents, which complicate inferences about sequence. **Table 3** links the principal therapeutic strategies with their biological rationale and the remaining translational question.

**Table 3:** Therapeutic strategies supported by retinoid biology and priorities for translational testing.

Strategy	Biological rationale	Supporting evidence	Priority comparison or endpoint	Refs.
Myeloid differentiation	Reduce functionally defined myeloid suppression	ATRA-treated mouse models; human blood PD	Partner regimen ± ATRA with paired blood–tumour suppression assays	(3–5, 57)
Vaccine priming	Remove host suppression or inhibit a tolerogenic DC circuit	Mouse vaccination, schedule experiments, SCLC immune PD; ALDH1A2 inhibition	Systemic ATRA versus cell-restricted RA-axis perturbation; defined priming window	(6, 13, 99)
Tumour recognition	Induce RNA sensing, antigen presentation or target ligands	Tumour-cell and mouse studies; divergent PD-L1 effects	Matched target engagement, killing and checkpoint-dependency assays	(9, 10, 15, 48)
Effector-cell conditioning	Promote effector differentiation or preserve function under exhaustion pressure	RARα-manipulated cytotoxic T cells; ATRA-conditioned CD8 and Vδ2 models	Receptor manipulation versus ligand conditioning; differentiation, persistence and killing	(16, 81, 85)
Combination with local therapy	Alter the myeloid response to local therapy	ATRA–radiotherapy and other local-treatment models	Timing and component controls with early on-treatment tumour sampling	(64, 100, 101)
Stromal reprogramming	Alter stellate-cell state and access to tumour nests	KPC spatial CD8 redistribution; stellate-cell mechanics; STARPAC exploratory PD	Chemotherapy ± ATRA plus spatial and functional tissue endpoints	(11, 91, 94)
Targeted delivery and metabolism	Reach the intended cells for a defined duration	Co-delivery, mucosal vaccination, CYP26A1 targeting and timed local release	Free ATRA, empty carrier and partner-cargo controls with cell-level exposure	(32–34, 107)

Strategies may overlap within a treatment programme. Experimental endpoints include immune function and tumour response; immune perturbations assess the contribution of immunity to tumour control. Human pharmacodynamics refers to biological responses in the sampled compartment. Incremental clinical benefit denotes the effect of adding ATRA to the same partner regimen. ATRA, all-trans retinoic acid; RA, retinoic acid; DC, dendritic cell; PD, pharmacodynamics; SCLC, small-cell lung cancer; KPC, Kras/Trp53-mutant pancreatic-cancer mouse model; ALDH1A2, aldehyde dehydrogenase 1 family member A2; CYP26A1, cytochrome P450 family 26 subfamily A member 1; PD-L1, programmed death-ligand 1.

## 5 Human pharmacodynamics and clinical evidence

### 5.1 Clinical pharmacodynamic activity

Human studies provide three complementary types of information: pharmacodynamic responses, clinical outcomes of the complete regimen, and the design and status of registered trials. **Table 2** separates these categories; **Supplementary Tables S1** and **S2** provide the detailed registry and clinical context. The sampled compartment is central to interpreting pharmacodynamics, because blood, tumour tissue, imaging and ex vivo assays capture different aspects of treatment activity.

Early retinoid–IFN programmes established dosing and pharmacokinetic experience with intermittent ATRA in paediatric refractory cancer (28). Phase II studies subsequently examined activity in renal-cell and squamous carcinomas (29, 30), including a randomised comparison of retinoid–IFN regimens in recurrent cervical cancer (108). Liposomal ATRA entered phase I evaluation with IFNα2b in advanced renal-cell carcinoma (109), while conventional ATRA–IFN treatment underwent clinical and pharmacokinetic study in cervical cancer (110). A subsequent phase I/II study extended the renal-cell programme to weekly liposomal ATRA (111). Collectively, these studies established tolerability and exposure experience, with limited or mixed clinical activity. In renal-cell carcinoma, Mirza and colleagues linked achieved ATRA exposure to circulating immature myeloid-cell changes and improved ex vivo immune competence, including allogeneic stimulation and recall responses. Subsequent IL-2 treatment altered the myeloid response (5). This sequential study established that clinically achieved exposure can modify circulating immune-cell biology and also demonstrated the influence of the partner treatment.

The small-cell lung cancer dendritic-cell vaccine programme measured circulating MDSCs and p53-specific immunity. Its interim and final reports describe a linked study family: an initial three-arm randomised stage of 55 patients followed by 14 additional patients in the vaccine–ATRA arm (6, 112). Among immune-evaluable patients, p53 responses occurred in 10/23 receiving vaccine–ATRA and 3/15 receiving vaccine alone. These antigen-specific measurements extend the biological interpretation beyond a change in myeloid frequency.

Melanoma studies extended blood-based assessment to checkpoint therapy. A small randomised ipilimumab pilot compared peripheral myeloid and effector responses with and without ATRA, and a subsequent single-arm pembrolizumab–ATRA study measured circulating MDSCs in 24 treated patients (17, 113). The circulating changes locate a pharmacodynamic response in blood. Their interpretation depends on differentiation, redistribution, sample handling, partner-treatment effects and changing disease burden. Paired tissue sampling with suppression assays would connect this systemic response to myeloid function within the tumour.

STARPAC examined pancreatic stroma using ATRA pharmacokinetics, diffusion-weighted MRI and stromal or soluble biomarkers. Twenty-seven patients received ATRA with gemcitabine and nab-paclitaxel; 19 received the recommended phase II dose, and 15 met the efficacy-evaluable definition at that dose (94). PTX3 and subsequent soluble-mediator analyses extend observations from the same programme (95, 96). These measurements characterised exploratory stromal pharmacodynamics. Other treatment-linked tissue studies demonstrated the feasibility of serial assessment of tumour biology. The breast-cancer study used preoperative treatment, whereas the prostate programme examined paired pre- and post-treatment biopsies in sequential trials of ATRA and of 13-cis-retinoic acid plus IFN (114, 115).

Blood-based studies reveal several components of the response to ATRA. A change in circulating myeloid frequency describes population composition; an ex vivo suppression or recall assay measures a functional property of sampled cells; a p53-specific response addresses immunity to the vaccine antigen (5, 6, 112). These observations occupy different positions between drug exposure and tumour control. Connecting these complementary measurements to tumour localisation, target recognition and killing would identify the steps through which systemic immune activity reaches the tumour.

### 5.2 Clinical outcomes and attribution of benefit

The pembrolizumab–ATRA trial reported a 71% objective response rate, including 50% complete responses, in 24 anti-PD-1-naive patients with metastatic melanoma. ATRA was administered for three days around each of the first four pembrolizumab cycles (17). Follow-up of the same cohort estimated five-year overall and progression-free survival at 54.7% and 36.1%, respectively. Median progression-free survival was updated from 20.3 to 13.8 months after an additional progression event in a patient with previously short follow-up (18). The follow-up establishes durability of the observed combination activity within this cohort.

Attributing that activity to ATRA requires a concurrent pembrolizumab-alone comparator. The cohort was small, most patients had normal baseline lactate dehydrogenase, and subsequent therapies affect the interpretation of overall survival (17, 18). Eligibility, disease burden, melanoma subtype and assessment schedules also complicate historical comparisons. In anti-PD-(L)1-refractory melanoma, the ATRA-containing KEYMAKER-U02A cohort did not meet its prespecified expansion criterion (19). This less favourable result limits generalisation across treatment settings, although differences between the regimens and populations preclude a direct estimate of the effect of prior checkpoint exposure.

The SCLC programme demonstrates a further distinction between biological activity and clinical benefit. The higher frequency of antigen-specific responses with vaccine–ATRA was not accompanied by improvement in the principal salvage-chemotherapy response endpoint or a clear survival advantage (6, 112). In STARPAC, median overall survival was 11.7 months in the 15 efficacy-evaluable patients treated at the recommended dose (94). That selected, uncontrolled estimate describes the combination; the randomised STARPAC2 trial was designed to assess the addition of ATRA to chemotherapy in locally advanced disease (116, 117).

Comparative chemotherapy evidence provides a separate clinical benchmark. A double-blind trial randomised 108 patients with hepatocellular carcinoma and extrahepatic metastases to FOLFOX4 plus ATRA (n = 53) or placebo (n = 55). The intention-to-treat analysis reported an overall-survival hazard ratio of 0.56 (95% CI 0.33–0.93) and a progression-free-survival hazard ratio of 0.62 (95% CI 0.41–0.94), favouring ATRA (118). Exploratory molecular analyses examined response-associated proteins. Paired immune measurements in a comparable randomised design could test whether changes in tumour immunity mediate the reported treatment effect.

Safety findings likewise belong to their respective regimens and reporting periods. Two STARPAC patients experienced dose-limiting grade 4 thrombocytopenia (94). The melanoma follow-up reported five patients with grade 3–4 pembrolizumab-attributed immune-related adverse events and one patient with a previously reported ATRA-attributed grade 4 event described as meningitis/stroke (18). Larger comparative studies are needed to characterise uncommon toxicity and the benefit–risk balance of adding ATRA.

A 2026 conference report from the atezolizumab–bevacizumab–ATRA study in refractory microsatellite-stable colorectal cancer described preliminary safety experience in 16 enrolled patients. Five required treatment interruptions and one required ATRA dose reduction; efficacy and biomarker results were still forthcoming (119).

The 2025 KEYMAKER-U02B update adds a first-line comparison to the refractory-disease findings. In this rolling-arm platform, 49 patients received favezelimab/pembrolizumab and 48 received the same combination plus ATRA. At a median follow-up of 6.6 months in both arms, each had an objective response rate of 33% (95% CI 20–48), and grade 3–5 treatment-related adverse events occurred in 27% of each arm (120). Together with the refractory U02A result, these data favour testing a prespecified retinoid-responsive mechanism within a defined partner regimen, with patient selection linked to the proposed cellular response (19, 120).

The next clinical comparison can therefore be designed around a specific mechanism already observed in patients or experimental models. For a myeloid-directed programme, an early sampling point could pair circulating-cell measurements with tumour myeloid composition and suppressive activity. For a stromal programme, tissue assessment could connect the imaging response with stellate-cell state and the position of CD8-positive cells relative to tumour nests. A prespecified later assessment would then relate these early changes to clinical outcome. Applying the same sampling schedule to the partner-only arm would separate changes associated with adding ATRA from those accompanying treatment more generally. This design builds directly on the blood pharmacodynamics of the melanoma studies and the stromal measurements of STARPAC (17, 94, 113). It would also allow a biologically active dose or schedule to be selected according to the intended cellular response, bringing pharmacology and immune mechanism into the clinical comparison.

### 5.3 Registered clinical development

Registered development spans chemotherapy- and checkpoint-containing regimens. STARPAC2 randomises pancreatic-cancer chemotherapy with or without ATRA (117). Checkpoint studies include non-small-cell lung cancer, IDH-mutant glioma and pancreatic cancer (122–124), alongside two triple-negative breast cancer programmes (125, 126) and studies in microsatellite-stable colorectal cancer and leiomyosarcoma (127, 128). A further pancreatic programme combines ATRA with SDK002, chemotherapy and anti-PD-1 therapy (129). **Supplementary Table S1** provides status, enrolment, update dates and structured-results availability for ten records checked on 21 September 2026.

The liposomal ATRA formulation HF1K16 is being evaluated in the dose-escalation study NCT05388487 (121). An AACR 2025 abstract described serial blood phenotyping in solid-tumour and glioma patients (130). A subsequent SITC report from the same programme included 21 patients with recurrent or refractory glioma and reported declining circulating MDSCs, recovery of lymphocyte counts and T-cell infiltration in one tumour specimen obtained after five treatment cycles (131). These blood and tissue observations extend formulation-dependent pharmacodynamic assessment to glioma.

## 6 Discussion

The central implication of the evidence is that the therapeutic value of ATRA depends on whether the cells it modifies control a reversible barrier to antitumour immunity. Differentiation of a suppressive myeloid population, activation of tumour-cell RNA sensing and preservation of CD8-positive T-cell function each provide a distinct route to benefit (3, 4, 15, 16). Local RA also sustains tolerogenic antigen-presenting-cell states (12, 13). Systemic treatment can therefore encounter both responsive effector pathways and counter-regulatory circuits within the same tissue. A treatment hypothesis should identify the function that limits response to the partner therapy and the cells in which ATRA can change it.

This interpretation also makes context dependence experimentally falsifiable. Apparent disagreement between studies may arise from different exposures, different recipient states or different endpoints. Cell-specific perturbations and direct schedule comparisons can identify which of these determinants accounts for a divergent response. The clearest examples are local synthesis circuits and vaccination schedules, where the manipulated source or timing changes the biological response (12, 13, 99). Chromatin perturbation, receptor manipulation and RA-associated transcriptional memory now provide additional functional examples (38, 78, 81). Their common implication is that a response profile should include both the current state and its inducibility: a baseline signature identifies the candidate population, while a controlled exposure establishes which immune function can change.

Clinical translation depends on how the induced biological response contributes to tumour control. Blood MDSC changes, stromal imaging signals and inflammatory transcriptional programmes locate treatment activity at different steps between exposure and tumour control. The SCLC experience is particularly instructive because improved antigen-specific responses coexisted with an unimproved principal clinical endpoint (112). Translation should therefore link three observations within the same study: exposure in the relevant compartment, recovery of the proposed immune function and an incremental effect of treatment. A short ATRA lead-in with paired blood and tumour sampling could help separate its early biological effects from those of the partner regimen. A randomised comparison against that same regimen would address clinical benefit.

Biomarker development should distinguish a cell’s capacity to respond from the tumour’s dependence on that response. CRABP2/FABP5 balance, receptor regulation and chromatin may identify retinoid-responsive cells; suppressive function, deficient recognition or stromal sequestration identifies the immune problem that treatment is intended to correct (35, 36, 43, 91). The strongest therapeutic opportunity is their intersection: a population that limits immunity and responds to clinically achievable retinoid exposure. A practical enrichment strategy would therefore combine evidence of the limiting immune process with demonstrable reversibility by the proposed intervention. This shifts selection from a single marker towards a biologically explicit treatment hypothesis.

A further priority is to resolve how responses in different compartments combine within an individual tumour. Pancreatic studies provide a concrete starting point: stellate-cell reprogramming and altered T-cell localisation coexist in the literature with variable epithelial responses associated with CRABP2 and FABP5 (11, 43, 91). Studying these processes together could identify tumours in which stromal access and epithelial susceptibility improve in parallel, as well as settings better suited to compartment-selective delivery. The same principle applies to vaccination, where differentiation of suppressive host myeloid cells and interruption of an autocrine RA circuit in vaccine dendritic cells offer complementary routes to priming (3, 13). We therefore favour developing ATRA combinations around a dominant, measurable immune constraint, while following the most plausible counter-regulatory compartment. This approach gives biological selectivity a practical meaning: the desired response is defined at the cellular level, and drug delivery, schedule and partner treatment are chosen to sustain it.

Discordant pharmacodynamic and clinical findings can guide the next intervention by locating the stage at which treatment activity is lost. Adequate exposure followed by a stable cellular state points towards receptor competence or patient selection; a phenotypic response accompanied by weak immune activity points towards effector availability or function. Persistent tumour growth despite improved immune function directs attention to recognition, tissue access and alternative resistance pathways. The SCLC and stromal programmes provide concrete settings for testing these relationships (94, 112). This sequence turns discordance into a basis for choosing the next mechanistic comparison.

Selectivity may ultimately be as important as exposure intensity. Targeted delivery or ex vivo lymphocyte conditioning could concentrate a favourable response in a chosen population, whereas systemic dosing may be preferable when coordinated myeloid and stromal effects are required. These approaches offer complementary ways to match exposure to the cellular mechanism of interest (16, 31–33).

## 7 Conclusion

ATRA can alter immune suppression, tumour recognition, effector function and stromal organisation through several molecular mechanisms. Its promise in solid-tumour immunotherapy lies in matching those effects to a defined cellular state and therapeutic setting. Human studies demonstrate pharmacodynamic activity and the feasibility of combining ATRA with immunotherapy or stromal treatment. The next stage is to establish which of these cellular effects adds clinical benefit to the partner regimen. Mechanistically selected combinations, functional tumour sampling and controlled comparisons can connect retinoid-responsive biology to a useful treatment strategy.
