# Supplementary material for: All-trans retinoic acid in solid-tumour immunity: context-dependent signalling, cellular states, and combination therapy
Source: Front Immunol. 2026 Oct 20;17:1953814. doi: 10.3389/fimmu.2026.1953814 (PMC13728497; doi:10.3389/fimmu.2026.1953814)
Supplement: Supplementary file 1 [file Table1.docx]

Supplementary tables

**Supplementary Table S1. Status of selected registered ATRA-containing clinical studies as of 21 September 2026.**

| **Identifier** | **Population / combination** | **Registry status** | **Enrolment** | **Last posted update** | **Results posted** |
| --- | --- | --- | --- | --- | --- |
| NCT04241276 | Locally advanced PDAC; chemotherapy ± ATRA | Active not recruiting | 170 (estimated) | 2026-01-07 | No |
| NCT05388487 | Refractory solid tumours; liposomal ATRA (HF1K16) | Unknown | 54 (estimated) | 2024-01-16 | No |
| NCT04919369 | Advanced NSCLC; atezolizumab + ATRA | Completed | 18 (actual) | 2026-07-20 | No |
| NCT05345002 | Recurrent IDH-mutant glioma; PD-1 inhibition + ATRA | Recruiting | 55 (estimated) | 2026-02-20 | No |
| NCT05482451 | Pancreatic cancer; nivolumab + ATRA | Unknown | 10 (actual) | 2024-08-21 | No |
| NCT06371274 | Advanced TNBC; toripalimab + ATRA | Recruiting | 29 (estimated) | 2025-08-12 | No |
| NCT06636981 | Advanced TNBC; toripalimab/chemotherapy + ATRA | Recruiting | 129 (estimated) | 2024-12-13 | No |
| NCT05999812 | Refractory MSS CRC; atezolizumab/bevacizumab + ATRA | Active not recruiting | 22 (actual) | 2026-06-17 | No |
| NCT06528769 | Advanced leiomyosarcoma; cemiplimab + ATRA | Recruiting | 16 (estimated) | 2025-09-30 | No |
| NCT07348107 | Advanced PDAC; SDK002, chemotherapy and anti-PD-1 + ATRA | Not yet recruiting | 10 (estimated) | 2026-01-16 | No |

Source: ClinicalTrials.gov individual study records, https://clinicaltrials.gov/study/ followed by the identifier. Status labels follow the registry; estimated enrolment denotes a planned value. The results column records structured registry results. Separate publications include AACR and SITC abstracts from HF1K16, a preliminary MSS colorectal safety abstract and the STARPAC2 protocol. The colorectal abstract covers 16 patients at an earlier cutoff; the registry records 22 actual participants. Abbreviations are defined in the main manuscript.

**Supplementary Table S2. Additional clinical context and interpretation of linked study reports.**

| **Study family** | **Population / reporting unit** | **Measurements or findings** | **Interpretation** |
| --- | --- | --- | --- |
| Historical IFN programmes | Multiple small studies; conventional/liposomal ATRA and other retinoids | Dose, schedule and PK experience; mixed clinical activity | Early dose and exposure studies used different retinoid isomers and oral/liposomal schedules. Original references: Adamson 1997; Escudier 1998; Weiss 1998; Goncalves 2001; Goldberg 2002; Boorjian 2007. |
| RCC ATRA–IL-2 | 18 patients; sequential intervention; Mirza 2006 | Exposure-linked blood myeloid and ex vivo immune changes | The sequential design captures ATRA-associated blood activity and subsequent IL-2-associated changes; survival comparisons used exposure-defined subgroups. |
| SCLC vaccine study family | 55 in initial three-arm stage; 14 added to vaccine–ATRA; 69 total | p53 responses: 10/23 immune-evaluable in vaccine–ATRA versus 3/15 vaccine alone | Iclozan 2013 reports interim immune findings; Chiappori 2019 reports final outcomes and the expanded combination arm of the same programme. The principal salvage-response endpoint was not improved. |
| Ipilimumab–ATRA | Small randomised pilot; Tobin 2018 | Peripheral MDSC and effector measurements | Randomised comparison of peripheral pharmacodynamics in a small pilot cohort. |
| Pembrolizumab–ATRA | 24 treated anti-PD-1-naive patients; Tobin 2023 and Borgers 2025 | ATRA 150 mg/m²/day for three days around the first four pembrolizumab cycles at the recommended dose. ORR 71%, CR 50%; updated median PFS 13.8 months. Five-year OS 54.7% (95% CI 36.4–82.1) and PFS 36.1% (95% CI 21.0–62.2). | Follow-up of the original single-arm cohort. Median PFS was updated from 20.3 to 13.8 months after a further progression event in a patient with previously short follow-up. The initial best CR rate and the proportion remaining in CR at later follow-up describe different outcomes. |
| KEYMAKER-U02A | Anti-PD-(L)1-refractory melanoma; Dummer 2024 conference abstract | 20 patients; no objective responses; expansion criterion not met | No observed objective responses in refractory disease; population and regimen differ from the anti-PD-1-naive programme. |
| KEYMAKER-U02B | First-line advanced melanoma; 2025 conference abstract; 49 fave/pembro and 48 fave/pembro + ATRA | ORR 33% (95% CI 20–48) in each arm; grade 3–5 treatment-related AEs 27% in each; median follow-up 6.6 months | No increase in reported ORR with ATRA at this early analysis of the rolling-arm platform. Response durability and survival are separate endpoints. Population and partner regimen differ from U02A. |
| STARPAC | 27 treated; 19 at recommended dose; 15 efficacy-evaluable; Kocher 2020 | ATRA 45 mg/m²/day on days 1–15/cycle with gemcitabine/nab-paclitaxel; PK, MRI and soluble markers | Median OS 11.7 months in 15 efficacy-evaluable patients. Grade 4 thrombocytopenia was dose-limiting in two patients. Subsequent biomarker publications analyse the same treatment programme. |
| HF1K16 | NCT05388487 dose-escalation study; AACR and SITC 2025 abstracts; SITC update includes 21 recurrent/refractory glioma patients | Serial blood MDSC and lymphocyte measurements; T-cell infiltration in one tumour specimen after five cycles. Survival reported separately for eight grade 4 and nine evaluable grade 2–3 glioma patients. | SITC extends the AACR report from the same study. Survival observations are uncontrolled and reported by tumour-grade subgroup. |
| FOLFOX4–ATRA | HCC with extrahepatic metastases; 108 randomised (53 ATRA, 55 placebo) | ITT OS HR 0.56 (95% CI 0.33–0.93); PFS HR 0.62 (0.41–0.94) | Double-blind comparison on the same chemotherapy backbone. Reported HRs favour ATRA; exploratory molecular profiling addressed response-associated proteins. |
| Atezolizumab–bevacizumab–ATRA | Refractory MSS CRC; NCT05999812; Ozer 2026 safety abstract; 16 enrolled | Five treatment interruptions; one ATRA dose reduction; efficacy and biomarker data forthcoming. | Preliminary safety analysis. The 16-patient abstract precedes the registry’s 22-patient enrolment update. |

Source articles are listed in the main manuscript bibliography. Additional identifiers: Mirza, doi:10.1158/0008-5472.CAN-06-1690; Chiappori, doi:10.1007/s00262-018-2287-9; Tobin 2018, doi:10.1016/j.intimp.2018.08.007; Tobin 2023, doi:10.1158/1078-0432.CCR-22-2495; Borgers, doi:10.1177/17588359251349321; Kocher, doi:10.1038/s41467-020-18636-w; Dummer, doi:10.1200/JCO.2024.42.16_suppl.9506; Zheng, doi:10.1158/1538-7445.AM2025-LB205. Gogas 2025, doi:10.1016/j.annonc.2025.08.2249; Sun 2023, doi:10.1038/s41392-023-01604-3; Ozer 2026, doi:10.1200/JCO.2026.44.2_suppl.114. Zheng, SITC 2025 update, doi:10.1136/jitc-2025-SITC2025.1350.
